# Medical Reports on Hospital Patients

**Published:** 1915-04-24

**Authors:** 


### PROBLEMS IN ADMINISTRATIVE MEDICINE.

### Medical Reports on Hospital Patients.

The question of the communication of professional information relative to patients under treatment in hospital, in consequence of the operation of the Employers' Liability Act and the National Insurance Act, has become a very practical and acute issue. Under these Acts claims for compensation are frequent, and in not a few instances the person making the claim has come under hospital observation. Medical evidence in such circumstances is often or even usually of the highest importance, and interested parties naturally wish to obtain it. One of the interests involved is, of course, the person or persons against whom the claim is advanced; and, in practice, this often means an accident insurance society. No one questions for a moment that such a society has a reasonable claim for medical information bearing on an issue which may mean serious financial liability. Indeed, this was well illustrated in a recent appeal to the House of Lords, which affirmed by a unanimous judgment the right of an employer to have, if he so desired, more than one medical examination of a workman claiming compensation on the ground that he was incapacitated for work as the result of an injury received in the course of his employment. Here there is a well-defined position of which hospitals must take due note; manifestly it would be unseemly for institutions claiming existence in the public interest to oppose or to obstruct proceedings which have legislative sanction. On the other hand, it has to be recognised that a patient is not less a patient because he is treated at a hospital. He is therefore under the care of a medical practitioner whose bounden duty it is to protect his interests and to preserve his secrets. The professional obligation to respect the patient's confidence is imperative, and to forget this or to ignore it is both a legal and a moral offence. There are thus two commanding principles to be recognised by the hospital authorities. The one, that medical examination in the circumstances here contemplated has legal sanction; the other, that no medical officer must be asked to report facts or to express opinions without the full knowledge and consent of the patient concerned.

The governors of St. Bartholomew's Hospital have recently had this question of reports on patients * under consideration, and have formulated certain rules for the benefit and direction of members of the junior staff. These rules affirm the confidential character of the hospital records and of all other information gained about a patient, whether by examination or by other means. From this it follows that access to such records can only be obtained on the application or with the consent of the patient or of his authorised representative, and such application or consent ought to be in writing. Acting on the same principle, it is ruled that no patient in the hospital can be examined by a medical practitioner or other person, acting on behalf of any outside body, without his consent. Further, it is essential that this consent shall be an intelligent one; that is, it shall be given or refused after full explanation afforded by an authorised official of the hospital. All this is in harmony with the principle of medical confidences, but it is well to have it stated authoritatively for the guidance of junior and inexperienced men. A useful hint, too, is given on the meaning and force of a subpœna. This, of course, compels attendance in Court, but it does not justify or compel the giving of information to a solicitor or other person serving the subpœna; here, again, to give information about a patient apart from his consent would constitute a breach of professional duty. The governors, perhaps wisely, have not entered on the difficult question of the extent of such professional duty as applying to a practitioner actually in the witness-box. No doubt the general rule is that any question sanctioned by the Court must be answered at the risk of a committal for contempt. At the same time, medical witnesses have on several occasions successfully appealed against a question on the ground that to answer it would be to break the seal of professional confidence; and even when such an appeal is refused it has the advantage of showing to the public the reluctance of the practitioner to reveal his patient's secrets, and puts the responsibility for the proceeding where it really rests—that is, with the law and the lawyers. This, however, is a delicate matter, and can hardly arise in cases in which resident medical officers are likely to be engaged. The one exception perhaps is in instances in which a patient whose life is insured dies in hospital and the insurance company concerned is inclined to set up a plea that the patient was suffering from disease at the date the insurance was effected. These experiences are not common, but they occur every now and again, and probably the best advice to a resident officer is to recommend him to give no information on the matter except after consultation with his chief. An excellent direction in the rules now before us is that all applications for reports must be referred to the clerk of the hospital, who will see that the request is properly authorised and the patient's consent duly obtained.

On one point we may venture a critical remark. The governors, while expressing the absence of objection to reports on patients, provided these are granted under the rules, stipulates that the report must be one which involves "an expression of opinion on the case," and does not "consist merely of statements of fact." This may lead to some difficulty as to the meaning of the term "fact."

It is common in medical reports when a conclusion is evident or beyond contest, or is near this position, to state the conclusion as a "fact"; and in view of this practice the rules with which we are now concerned are in this respect not quite free from ambiguity. But their general tenor is excellent, and we are sure they will be helpful.

* For text of regulations see p. 90.

